# Long-term clinical course and progression of lymphangioleiomyomatosis in a single lung transplant referral centre in Korea

**DOI:** 10.1038/s41598-022-12314-1

**Published:** 2022-05-18

**Authors:** Shihwan Chang, Ji Soo Choi, Ah Young Leem, Su Hwan Lee, Sang Hoon Lee, Song Yee Kim, Kyung Soo Chung, Ji Ye Jung, Young Ae Kang, Young Sam Kim, Jin Gu Lee, Hyo Chae Paik, Hyo Sup Shim, Eun Hye Lee, Moo Suk Park

**Affiliations:** 1 Division of Pulmonary and Critical Care Medicine, Department of Internal Medicine, Institute of Chest Diseases, Severance Hospital, Yonsei University College of Medicine, 50-1 Yonsei-ro, Seodaemun-gu, Seoul, 03722 Republic of Korea; 2 Division of Pulmonary, Allergy and Critical Care Medicine, Department of Internal Medicine, Yongin Severance Hospital, Yonsei University College of Medicine, 363 Dongbaekjukjeon-daero, Giheung-gu, Yongin, 16995 Republic of Korea; 3 Department of Thoracic and Cardiovascular Surgery, Severance Hospital, Yonsei University College of Medicine, 50-1 Yonsei-ro, Seodaemun-gu, Seoul, 03722 Republic of Korea; 4 Department of Pathology, Severance Hospital, Yonsei University College of Medicine, 50-1 Yonsei-ro, Seodaemun-gu, Seoul, 03722 Republic of Korea

**Keywords:** Medical research, Risk factors

## Abstract

We aimed to describe the clinical features of lymphangioleiomyomatosis (LAM) in Korean patients and identify factors associated with progressive disease (PD). Clinical features of 54 patients with definite or probable LAM from 2005 to 2018 were retrospectively analysed. Common features were pneumothorax (66.7%) and abdominal lymphadenopathy (50.0%). Twenty-three (42.6%) patients were initially treated with mechanistic target of rapamycin (mTOR) inhibitors. Lung transplantation (LT) was performed in 13 (24.1%) patients. Grouped based on the annual decline in forced expiratory volume in 1 s (FEV_1_) from baseline and LT, 36 (66.7%) patients exhibited stable disease (SD). All six deaths (11.1%) occurred in PD. Proportion of SD was higher in those treated initially with mTOR inhibitors than in those under observation (*p* = 0.043). Univariate analysis revealed sirolimus use, and baseline forced vital capacity, FEV_1_, and diffusing capacity of the lungs for carbon monoxide are associated with PD. Multivariate analysis showed that only sirolimus use (odds ratio 0.141, 95% confidence interval 0.021–0.949, *p* = 0.044) reduced PD. Kaplan–Meier analysis estimates overall survival of 92.0% and 74.7% at 5 and 10 years, respectively. A considerable proportion of LAM patients remain clinically stable without treatment. LT is an increasingly viable option for patients with severe lung function decline.

## Introduction

Lymphangioleiomyomatosis (LAM) is a rare systemic disease that primarily affects women of reproductive age^1^. The disease, characterized by proliferation of smooth-muscle-like LAM cells in lungs and lymphatic system, results in cystic lung destruction, chylothorax, renal angiomyolipoma, and formation of lymphangioleiomyomas. LAM can occur sporadically or in association with tuberous sclerosis complex (TSC). Clinical manifestations of LAM include exertional dyspnoea, haemoptysis, and pneumothorax. The disease progresses with a continuous decline in lung function, eventually resulting in clinically important respiratory impairment.

Because previous studies included a relatively small number of severe cases, LAM was considered a fatal disease affecting women primarily of child-bearing age^2^. Recent studies describe LAM as a chronic disease with a highly diverse clinical phenotype and a relatively long life expectancy^3–5^. Increased detection of asymptomatic or mild cases through regular health screenings and more frequent high-resolution computed tomography (HRCT) scans might account for this change. There is no definite evidence of racial or ethnic differences in incidence or clinical course of LAM^2^.

The clinical experience of LAM in Korea is in line with the above trend. A study of 21 patients in 1999 revealed that, despite hormonal treatment, the disease progressed in 80% of the patients and resulted in the death of 30% of the patients followed up for more than 12 months^6^. A more recent nationwide survey of 63 patients found an increase in the incidence of LAM since 2004, with a higher 5-year survival of 84%^7^. The study further showed that patients diagnosed before 2004 had higher rates of dyspnoea, lower baseline forced expiratory volume in 1 s (FEV_1_) and diffusing capacity of the lungs for carbon monoxide (DL_CO_).

Therapeutic options for LAM are limited. In the Multicentre International Lymphangioleiomyomatosis Efficacy and Safety of sirolimus (MILES) trial, sirolimus was found to stabilize the decline of lung function^8^. Subsequent studies found that sirolimus not only stabilizes lung function^9,10^, but also improves other clinical manifestations, such as chylous effusions and lymphangioleiomyomas^11^. Hormonal therapy has only shown limited efficacy in post-menopausal women^12^. In the advanced stages, lung transplantation (LT) remains as a definitive treatment option^13,14^.

Selecting the optimal treatment require a better understanding of LAM, especially the factors that affect disease progression. Some clinical factors, such as baseline lung function^3,15,16^, age at diagnosis^3,17,18^, and menopausal status^3,17^, were consistently thought to affect the natural course of LAM. Despite these recent findings, the causes of disease progression remain unclear. The aim of this study was to describe the clinical features and clinical course of LAM, and to identify factors that may be associated with rapid disease progression of LAM.

## Results

### Baseline characteristics

A total of 59 patients with LAM diagnosis on electronic medical records was identified from 2005 to 2018 at Severance Hospital, South Korea. All diagnoses of LAM have been reviewed based on the European Respiratory Society (ERS) guidelines^1^.

Among these patients, 5 patients who were excluded were diagnosed with ‘possible LAM’ based on the ERS guidelines. These 5 patients did not show any respiratory symptoms, clinical features indicative of LAM, or decline in lung function in spirometry, but only exhibited the typical features of LAM on HRCT. Two of these patients received video-assisted thoracic surgical biopsy, but with no evidences of LAM in the final pathology report.

**Figure 1:**
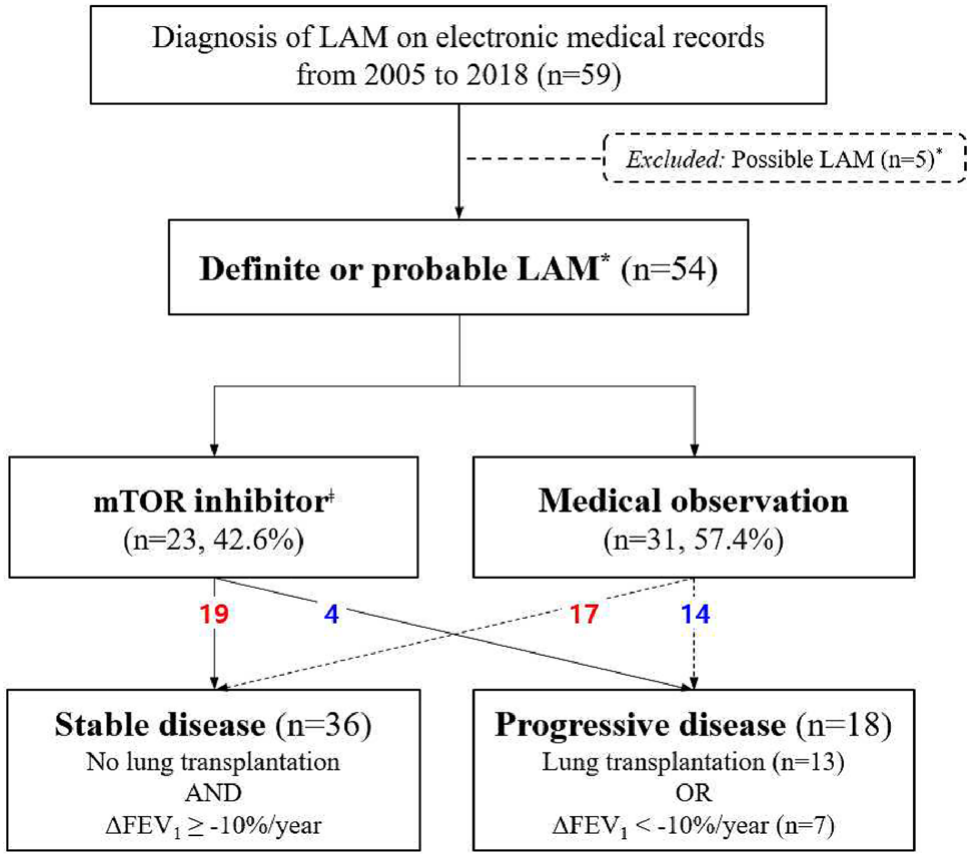
Flowchart of patient selection and study groups. Patients included in the final analysis are diagnosed with definite or probable LAM. These patients were divided by whether mTOR inhibitor was used as definitive treatment. Then, patients were subsequently divided by the disease course, according to lung transplantation status and ΔFEV_1,_ defined as annual decline in the percentage of the predicted FEV_1_ compared to baseline. *Diagnosis based on ERS guidelines (2010, ERJ) on LAM. ^ǂ^Three patients treated with everolimus had tuberous sclerosis complex-associated LAM and angiomyolipoma. Figure was created using Microsoft PowerPoint version 16.0 and Microsoft Paint version 11.2201. *LAM*, lymphangioleiomyomatosis; *mTOR*, mechanistic target of rapamycin; *FEV*_*1*_, forced expiratory volume in 1 s.

The final analysis was performed with a total of 54 patients diagnosed with definite (52/54, 96.3%) or probable (2/54, 3.7%) LAM. Regardless of the use of mechanistic target of rapamycin (mTOR) inhibitors, the study population was divided into two groups based on their disease course: namely, stable and progressive disease. Stable disease (SD) course was defined as annual decline of less than 10% in the percentage of the predicted FEV_1_ and not receiving LT; progressive disease (PD) course was defined as either annual decline of greater than or equal to 10% in the percentage of the predicted FEV_1_ or undergoing LT (Fig. 1). The annual decline in the predicted FEV_1_ was calculated in each patient by dividing the percent difference between the final and baseline predicted FEV_1_ by the time between the two pulmonary function test (PFT) measurements.

**Figure 2:**
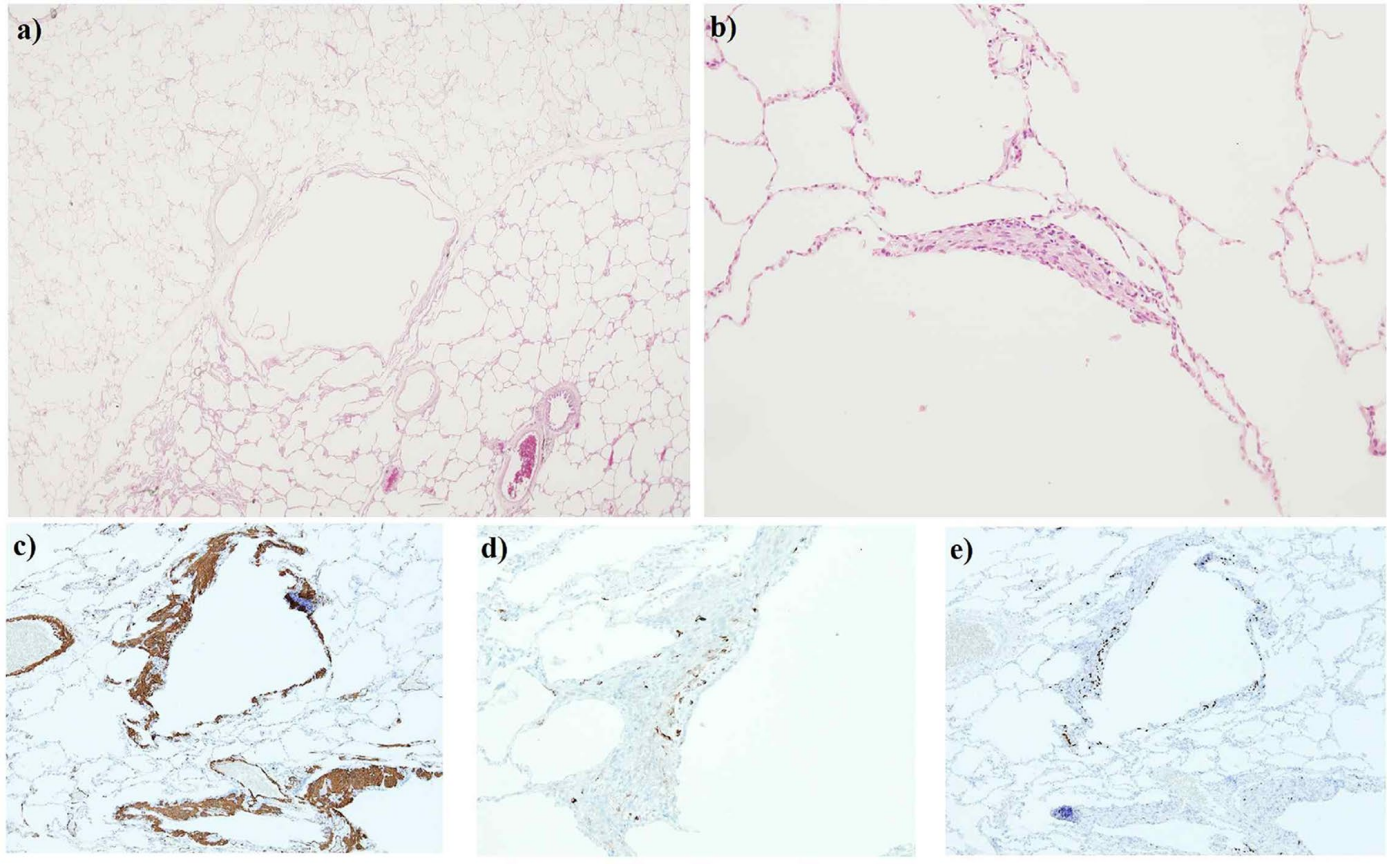
Lung biopsy from a patient diagnosed with lymphangioleiomyomatosis. Lung biopsy specimen from a patient diagnosed with lymphangioleiomyomatosis. Hematoxylin and eosin staining reveals (**a**) multiple cysts of varying sizes at low magnification (10X) and (**b**) spindle cells at high magnification (100X). Immunohistochemical staining shows positive stains for (**c**) smooth muscle actin, (**d**) HMB-45, and (**e**) progesterone receptor. Figure was created by arranging the panel images with Microsoft Paint version 11.2201.

Table 1 summarizes the baseline characteristics of the study population. All patients except one (53/54, 98.1%) were female, with a median age at diagnosis of 36.5 years. Only four (9.5%) patients were post-menopausal at the time of diagnosis. Six patients (11.1%) had TSC-associated LAM (TSC-LAM), one of whom was the only male patient in the study. In 36 (66.7%) patients, diagnosis of LAM was established with biopsy (Fig. 2). All patients showed characteristic patterns of LAM on HRCT. Pneumothorax was observed in 36 (66.7%) patients, abdominal lymphadenopathy in 27 (50.0%), angiomyolipoma in 18 (33.3%), and chylothorax in six (11.1%). LT was performed in 13 (24.1%) patients. mTOR inhibitors were used as first-line treatment in 23 (42.6%) patients. Sirolimus was administered in 20 (37.0%) patients; everolimus in three (5.6%) patients, all of whom had TSC-LAM.

**Table 1:** Baseline characteristics of 54 patients with lymphangioleiomyomatosis.

	All (*n* = 54)	Stable (*n* = 36)	Progressive (*n* = 18)	*p *value
Females	53 (98.1)	35 (97.2)	18 (100.0)	0.475
Age at diagnosis, years	36.50 [31.75–42.25]	37.50 [32.00–44.75]	35.00 [30.75–39.50]	0.340
Median follow-up, years	4.89 [1.53–8.43]	3.75 [1.19–8.41]	5.62 [2.12–9.00]	0.204
Menopause at diagnosis	4 (9.5)	3 (8.3)	1 (5.6)	0.082
Mortality	6 (11.1)	0 (0.0)	6 (33.3)	**0.001**
**Diagnosis**				0.547
Probable	2 (3.7)	2 (5.6)	0 (0.0)	
Definite	52 (96.3)	34 (94.4)	18 (100.0)	
Biopsy-proven	36 (66.7)	21 (58.3)	15 (83.3)	0.066
**Type of LAM**				1.000
TSC-LAM	6 (11.1)	4 (11.1)	2 (11.1)	
Sporadic LAM	48 (88.9)	32 (88.9)	16 (88.9)	
Characteristic HRCT	54 (100.0)	36 (100.0)	18 (100.0)	–
Pneumothorax	36 (66.7)	24 (66.7)	12 (66.7)	1.000
Abdominal LAP	27 (50.0)	15 (41.7)	12 (66.7)	0.083
Angiomyolipoma	18 (33.3)	15 (41.7)	3 (16.7)	0.066
Chylothorax	6 (11.1)	4 (11.1)	2 (11.1)	1.000
TSC gene mutation*	6/20 (30.0)	4/17 (23.5)	2/3 (66.6)	0.202
TSC-LAM	3/4 (75.0)	1/2 (50.0)	2/2 (100.0)	
Sporadic LAM	3/16 (18.8)	3/15 (20.0)	0/1 (0.0)	
**Initial treatment**				**0.043**
mTOR inhibitors	23 (42.6)	19 (52.8)	4 (22.2)	
Sirolimus	20 (37.0)	17 (47.2)	3 (16.7)	
Everolimus	3 (5.6)	2 (5.6)	1 (5.6)	
Medical observation	31 (57.4)	17 (47.2)	14 (78.8)	
Lung transplantation	13 (24.1)	0 (0.0)	13 (72.2)	
**Baseline PFT**				
FVC, % predicted^ǂ^	83.00 [69.50–90.25]	84.00 [76.00–91.00]	76.00 [45.50–90.00]	0.119
FEV_1_, % predicted^ǂ^	73.00 [46.50–91.00]	81.00 [67.50–92.00]	32.00 [21.00–63.00]	** < 0.001**
FEV_1_/FVC, % predicted^ǂ^	72.50 [46.75–83.00]	75.00 [71.50–84.50]	39.00 [31.50–63.00]	** < 0.001**
DL_CO_, % predicted^§^	59.50 [38.75–77.50]	71.00 [52.75–80.00]	30.50 [17.25–55.75]	**0.001**

All data expressed in n (%) or median [interquartile range]. *Data available for *n* = 20 in all patients, *n* = 17 in SD, *n* = 3 in PD. ^ǂ^Data available for *n* = 50 in all patients, *n* = 33 in SD, *n* = 17 in PD. ^§^Data available for *n* = 42 in all patients, *n* = 28 in SD, *n* = 14 in PD. *LAM*, lymphangioleiomyomatosis; *TSC*, tuberous sclerosis complex; *HRCT*, high-resolution computed tomography; *LAP*, lymphadenopathy; *mTOR*, mechanistic target of rapamycin; *PFT*, pulmonary function test; *FVC*, forced vital capacity; *FEV*_*1*_, forced expiratory volume in 1 s; *DL*_*CO*_, diffusion capacity of the lungs for carbon monoxide; *SD*, stable disease; *PD*, progressive disease. Significant *p*-values are in Bold.

**Figure 3:**
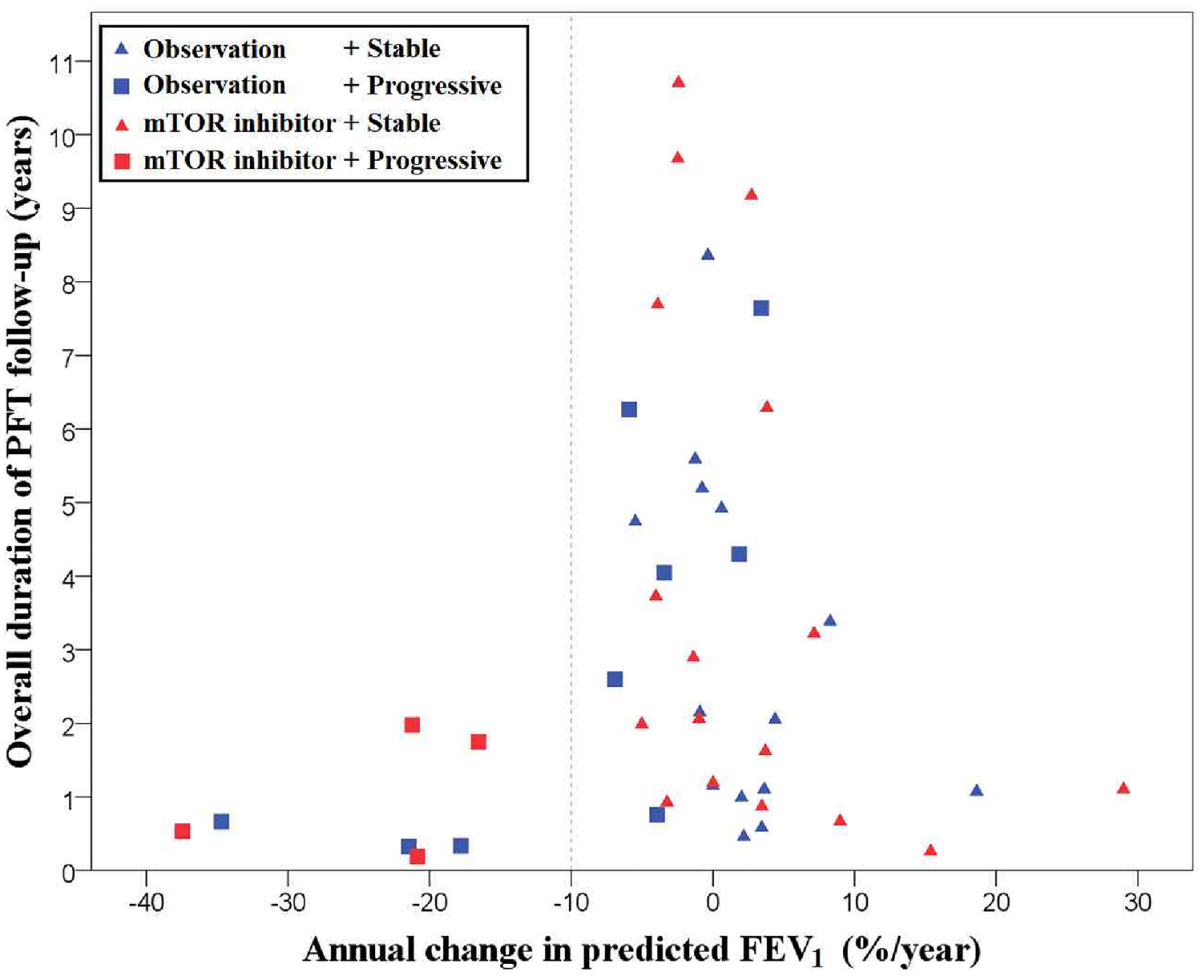
Distribution of annual FEV_1_ change and follow-up duration of pulmonary function test by patient groups. The annual decline in the percentage of the predicted FEV_1_ compared to baseline and the duration of follow-up of PFT for each patient is plotted, using SPSS software version 23 (www.ibm.com/analytics/spss-statistics-software) and Microsoft Paint version 11.2201. Patients are divided into four groups, according to mTOR inhibitor use and disease course. *mTOR*, mechanistic target of rapamycin; *PFT*, pulmonary function test; *FEV*_*1*_*,* forced expiratory volume in 1 s.

In the cohort, 36 (66.7%) patients showed SD course, 18 (33.3%) showed PD course. All 13 patients who received LT showed PD course. No significant differences in clinical features at diagnosis at were observed between two groups. However, the treatment methods and baseline PFT findings were significantly different between the groups. The proportion of patients exhibiting SD was higher in those initially treated with mTOR inhibitors than in whose who were under medical observation (mTOR inhibitor 19/23, 82.6% vs. medical observation 17/31, 54.8%, *p* = 0.043; Table 1, Fig. 3). All PFT parameters at baseline, except forced vital capacity (FVC), were significantly worse in patients with PD (*p* < 0.001 for FEV_1_, *p* < 0.001 for FEV_1_/FVC ratio, *p* = 0.001 for DL_CO_). Changes in predicted FEV_1_ values with respect to time are shown in Supplementary Figure S1.

**Figure 4:**
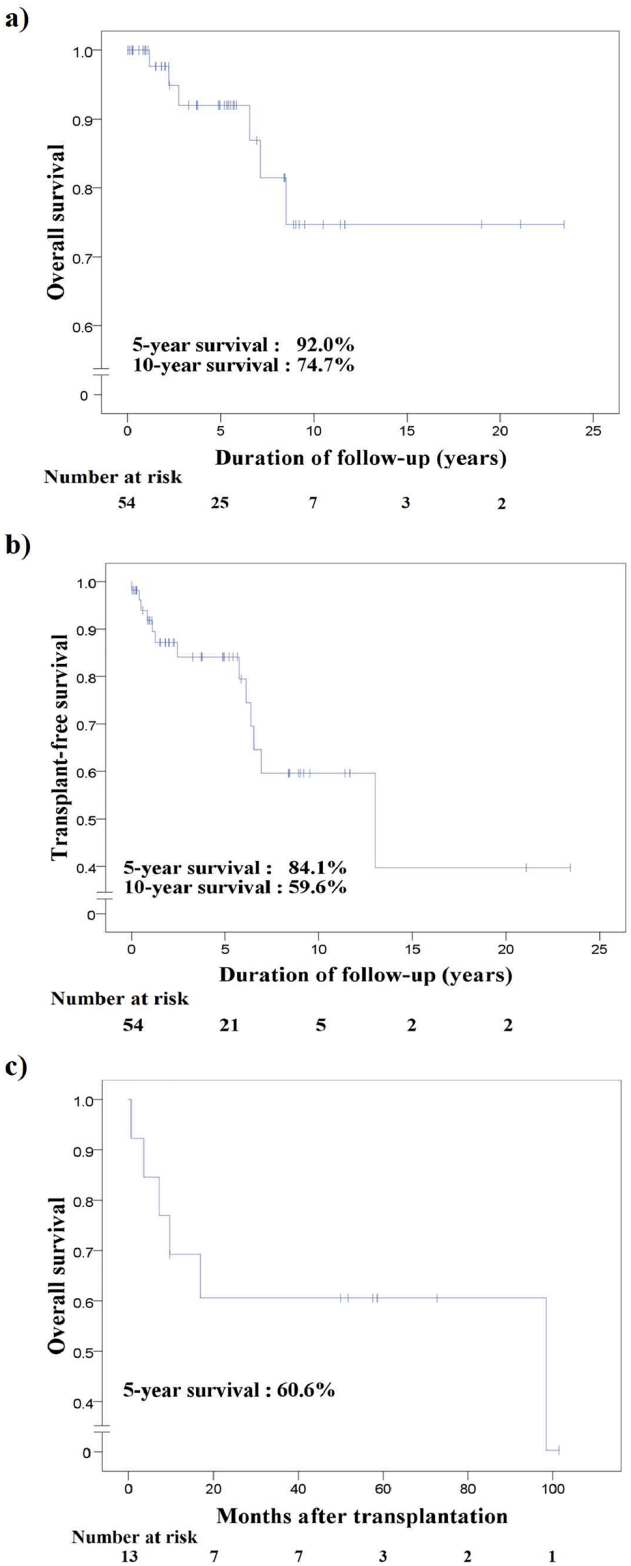
Kaplan–Meier analyses for survival. Kaplan–Meier analyses of (**a**) overall survival for all patients, (**b**) transplant-free survival for all patients, and (**c**) overall survival for lung transplantation. Figure was created using SPSS software version 23 (www.ibm.com/analytics/spss-statistics-software) and Microsoft Paint version 11.2201.

Six (11.1%) patients died during the follow-up period; all of them had undergone LT. Overall survival rates using the Kaplan–Meier analysis were 92.0% and 74.7% at 5 years and 10 years, respectively (Fig. 4a). Transplant-free survival rates were 84.1% and 59.6% at 5 years and 10 years, respectively (Fig. 4b).

### Factors associated with progressive disease

Logistic regression analyses were performed to determine factors associated with PD. The following clinical parameters were considered: age at diagnosis, menopause at the time of diagnosis, TSC-LAM, use of sirolimus, baseline PFT parameters, pneumothorax, abdominal lymphadenopathy, angiomyolipoma, and chylothorax (Table 2). Age at diagnosis (cut-off at 35 years) and baseline PFT parameters (cut-off at 70% of predicted value for all parameters) were analysed as categorical variables. Univariate analysis identified poor baseline PFT (odds ratio (OR) 8.667, 95% confidence interval (CI) 2.230–33.682, *p* = 0.002 for baseline FEV_1_) and medical observation (OR for use of sirolimus 0.224, 95% CI 0.055–0.908, *p* = 0.036) to be associated with PD. Multivariate analysis, performed with sirolimus use, baseline FVC, baseline FEV_1_, baseline DL_CO_, abdominal lymphadenopathy, and angiomyolipoma in the model, showed that only the use of sirolimus (OR 0.141, 95% CI 0.021–0.949, *p* = 0.044) reduces the risk of PD (Table 2). In an additional multivariate analysis performed with sirolimus use, baseline FEV_1_ (cut-off at 80% of predicted value), abdominal lymphadenopathy, and angiomyolipoma, both sirolimus use (OR 0.118, 95% CI 0.023–0.602, p = 0.010) and poor baseline FEV_1_ (OR 5.026, 95% CI 1.021–24.741, p = 0.047) were significantly associated with PD course (Supplementary Table S1, Model 2).

**Table 2:** Logistic regression analysis of factors associated with the progression of lymphangioleiomyomatosis.

Parameters	Univariate			Multivariate		
	OR	95% CI	*p *value	OR	95% CI	*p *value
Age at diagnosis > 35 years	0.509	0.162–1.601	0.248	–	–	–
Menopause	0.933	0.087–10.040	0.955	–	–	–
TSC-LAM	0.625	0.113–3.461	0.590	–	–	–
Use of sirolimus	0.224	0.055–0.908	**0.036**	0.141	0.021–0.949	**0.044**
FVC, ≤ 70% predicted	4.978	1.295–19.130	**0.019**	0.937	0.112–7.825	0.952
FEV_1_, ≤ 70% predicted	8.667	2.230–33.682	**0.002**	3.110	0.478–20.247	0.235
FEV_1_/FVC, ≤ 70% predicted	17.333	3.866–77.719	** < 0.001**	–	–	–
DL_CO_, ≤ 70% predicted	13.000	1.492–113.251	**0.020**	10.062	0.823–123.035	0.071
Pneumothorax	1.000	0.301–3.321	1.000	–	–	–
Abdominal LAP	2.800	0.858–9.139	0.088	1.310	0.219–7.849	0.768
Angiomyolipoma	0.280	0.069–1.142	0.076	0.441	0.065–3.009	0.403
Chylothorax	1.000	0.165–6.052	1.000	–	-	–

Progression of LAM is defined as receiving lung transplantation or ΔFEV_1_ less than − 10%/year, as described in Fig. 1. All data for PFT parameters use values at baseline. PFT parameters are categorical variables, with 70% of predicted value as cut-offs. The following variables are included in the model for multivariate analysis: use of sirolimus, predicted FVC, predicted FEV_1_, predicted DL_CO_, abdominal lymphadenopathy, and angiomyolipoma. *OR*, odds ratio; *CI,* confidence interval; *TSC*, tuberous sclerosis complex; *LAM*, lymphangioleiomyomatosis; *mTOR*, mechanistic target of rapamycin; *FVC*, forced vital capacity; *FEV*_*1*_, forced expiratory volume in 1 s; *DL*_*CO*_, diffusion capacity of the lungs for carbon monoxide; *LAP*, lymphadenopathy; *PFT*, pulmonary function test. Significant *p*-values are in Bold.

### Patients who underwent lung transplantation

Table 3 summarizes the clinical features of all patients who underwent LT. All patients were females diagnosed with sporadic LAM, with a median age of 37 years at disease onset. The most common feature observed was pneumothorax (n = 10, 76.9%) followed by abdominal lymphadenopathy (n = 9, 69.2%), angiomyolipoma (n = 2, 15.4%), and chylothorax (n = 1, 7.7%). All patients underwent bilateral transplantation, except one who underwent two unilateral transplantations. Recurrence of LAM, observed in two (15.4%) patients, was treated with sirolimus. As mentioned earlier, six patients (46.2% of LT cases) died: three due to pneumonia, one due to infection other than pneumonia, one due to cerebral infarction, and one due to an unknown cause. Kaplan–Meier analysis showed a median survival of 98.4 months (95% CI 0.0–217.1) and estimated 5-year survival of 60.6% (Fig. 4c). A higher proportion of patients who underwent LT after 2011 (5 out of 7, 71.4%) survived than those who underwent LT before 2011 (1 out of 5, 20.0%).

**Table 3:** Clinical features of patients with lymphangioleiomyomatosis who received lung transplantation.

Patient	Age at onset	LT year	Follow-up (months)		Pneumo﻿thorax	Abd. LAP	AML	Chylo﻿thorax	Recur	Death	Cause of death	Notes
			Before LT	After LT								
1	36	2004, 2012	5.0	98.4	+	+	−	−	−	+	Pneumonia	Two unilateral operations
2	33	2007	29.8	3.5	−	+	−	+	−	+	Cerebral infarction	
3	35	2008	10.2	16.9	−	+	−	−	−	+	Infection	
4	37	2009	70.1	9.8	−	−	−	−	−	+	Unknown	
5	48	2010	2.5	101.4	+	+	−	−	+	−	−	Sirolimus use, due to LAM recur
6	38	2011	79.5	7.2	+	+	+	−	−	+	Pneumonia	
7	26	2011	84.4	57.6	+	+	−	−	+	−	−	Sirolimus use, due to LAM recur
8	29	2011	13.6	0.6	+	−	−	−	−	+	Pneumonia	
9	31	2012	158.5	72.7	+	+	+	−	−	−	−	
10	39	2013	6.3	58.6	+	−	−	−	−	−	−	
11	41	2014	77.6	49.9	+	+	−	−	−	−	−	
12	42	2014	15.5	51.8	+	−	−	−	−	−	−	Sirolimus use, due to tacrolimus side effect
13	51	2017	74.5	9.8	+	+	−	−	−	−	−	
Total*	37	2011	70.1	49.9	76.9%	69.2%	15.4%	7.7%	15.4%	46.2%	−	−

All patients are female, diagnosed with sporadic LAM. LAM recurrences, identified in two patients, were both treated with sirolimus. *Values in the last row indicate the median values or incidences of each column. *LT*, lung transplantation; *AML*, angiomyolipoma; *Abd. LAP*, abdominal lymphadenopathy; *LAM*, lymphangioleiomyomatosis.

## Discussion

Our study described the long-term clinical course of 54 patients diagnosed with definite or probable LAM. Patients with SD were more likely to have better baseline PFT and to be treated with mTOR inhibitors. Multivariate analysis identified medical observation as the only factor increasing the risk of PD. As a tertiary referral centre, we included a high proportion of patients with LT, compared to previous studies^5,19^. Interestingly, we found that one-third of all patients (n = 17) in the study showed SD without definitive treatment, possibly suggestive of a phenotypic subtype of LAM.

In our study, sirolimus use was a significant predictor of SD course. Consensus statements recommend considering the use of sirolimus in patients showing rapid progression; however, the definition of rapid progression is not clear^1,20^. The efficacy of sirolimus has been demonstrated in patients with predicted FEV_1_ value of 70% or less^8^, and with symptomatic chylothorax^11^. Other studies have suggested that patients with rapidly declining FEV_1_, reduced DL_CO_, exercise-induced or resting hypoxemia, angiomyolipomas or lymphangioleiomyomas might benefit from sirolimus^21,22^.

Despite growing evidence of benefit of sirolimus treatment, our study shows a high proportion of patients who have not received sirolimus treatment. Its use in the treatment of LAM was approved for reimbursement by the Korean National Health Insurance starting early 2016, five years after results from the MILES trial were available. This temporal discrepancy, with a lack of clear consensus on the proper conditions for sirolimus use, might have led to a conservative use of sirolimus in this study. While we observe that the changes in predicted FEV_1_ for patients treated with sirolimus are variable among patients (Supplementary Figure S2), multiple models of regression analysis for predicting PD course suggests that sirolimus treatment reduces the risk for exhibiting PD course (Table 2, Supplementary Table S1). Additionally, we found that the proportion of patients exhibiting SD was higher in those initially treated with mTOR inhibitors than in those who were under medical observation. Sirolimus treatment should be strongly considered in patients exhibiting high-risk features, such as low baseline FEV_1_ and/or DL_CO_, symptomatic chylothorax, and hypoxemia.

A significant proportion (*n* = 14, 60.9%) of patients on mTOR inhibitors experienced adverse events (Supplementary Table S2), most common of which was oral mucositis or ulcer (*n* = 8, 34.8%), followed by infection (*n* = 4, 17.4%). Any event of drug discontinuation was documented in 12 (52.2%) patients, in seven of whom the drug was discontinued permanently. Rates of adverse events were comparable to those reported in other studies, although sirolimus drug level was maintained higher in our study^9,23^. These studies concluded that a lower dose of sirolimus was still effective in slowing FEV_1_ decline. As adverse events might eventually lead to treatment discontinuation, further studies are warranted to determine the optimal sirolimus dosage in LAM.

We also found that better baseline lung function is associated with stable course (Table 2). A study on mTOR inhibitor-naïve patients in Japan revealed that faster FEV_1_ decline was observed in the patient group with a baseline predicted FEV_1_ value of 70% or less^15^. The group with faster FEV_1_ decline not only had poor overall baseline PFT parameters, but also showed faster decline in FVC. In a large retrospective review of 275 patients, initial FEV_1_ and DL_CO_, in addition to age, were found to be predictors of lung function decline^16^. A longitudinal analysis of a LAM cohort identifies baseline PFT (FEV_1_ and DL_CO_) as most predictive factors of mortality^3^. In the MILES study, which only enrolled patients with FEV_1_ of 70% or less of the predicted value, faster FEV_1_ decline was seen in patients with bronchodilator response in spirometry^17^.

Consistent with many previous findings, our regression analysis shows that baseline predicted FVC, FEV_1_, and DL_CO_ of 70% or less are independently associated with PD course. While these PFT parameters were not found to be significant in multivariate analysis presented in Table 2, using higher cut-offs of 80% or less of predicted value for PFT parameters show that FEV_1_ is a significant predictor in PD course. In a multivariate regression analysis model containing sirolimus use, baseline FEV_1_ (cut-off at 80% of predicted value), abdominal lymphadenopathy, and angiomyolipoma, baseline FEV_1_ was found to be a significant predictor of PD course (Supplementary Table S1, Model 2). Subsequent Kaplan–Meier analyses of patients at risk for death and/or LT found a significantly higher frequency of death or LT in patients with poor initial FEV_1_, while no association was seen with initial DL_CO_ (Supplementary Figure S3 and S4). These findings indicate that FEV_1_ is more significant than other PFT parameters in determining the disease status.

Past studies have suggested different prognostic predictors for LAM. In addition to PFT abnormalities, older age at diagnosis^3,17,18^, menopausal status^3,17^, presence of pneumothorax^15^, chylous effusion^11^, and angiomyolipoma^18^, and use of supplemental oxygen and hormonal agents^18^ have been identified as clinical features associated with the disease progression and/or mortality due to LAM. However, our study found no other clinical features correlated with severity of the disease, likely due to the differences in study design. Another possible reason for this discrepancy is the relatively small size of the study population.

We observe a high proportion of patients with stable lung function in this cohort. Of the 31 patients in this cohort initially under medical observation, 17 exhibited SD. These patients, comprising nearly one-third of the study population, were associated with good baseline lung function, menopause at diagnosis, and absence of abdominal lymphadenopathy (Supplementary Table S3). Clinical manifestations of LAM are highly heterogeneous^21^. As seen in our study population (Supplementary Fig. 1a), lung function changes in patients with LAM have also been reported to vary widely, with one study reporting 42% of patients showing normal findings on spirometry^24^. Because the reasons for this variability are poorly understood, future studies are warranted to elucidate the benefit of sirolimus in patients with stable LAM phenotype.

Our study included 13 patients (24.1%) who underwent LT, which is higher than that in other observational studies^5,19^. All patients were female and diagnosed with sporadic LAM. Most of these patients had rapidly declining FEV_1_ (Fig. 3). LAM recurred in two patients, both of whom were treated with sirolimus. Similar to the observation in another study^14^, patients who underwent transplantation after 2011 in our study showed better survival outcomes than those who underwent transplantation before 2011 (Table 3). This was most likely due to the increased experience of transplantation team. LT remains a viable treatment option in LAM patients with significant decline in lung function.

Although LAM cohorts in earlier studies showed poor survival, recent studies suggest a more favourable prognosis for LAM. Survival rates at 10 years are reported to be 86%, 76%, and 79% across patients with LAM in the United States, Japan, and France, respectively^5,18,24^. Median transplant-free survival is over 20 years according to a recent LAM registry in the United States^3^. Consistent with these findings, the survival rate of 74.7% at 10 years in our study shows that LAM has a rather favourable prognosis.

There are some limitations to this retrospective single-centre study. Patients show a significant variation in the PFT values at baseline (Table 1, Supplementary Figure S1a), which may underestimate the effect of poor baseline PFT on subsequent disease course. However, to adjust for this issue, we add LT as another criterion for defining disease course. Of the 15 patients with severe airflow limitations (predicted FEV_1_ of less than 50%) at baseline, 12 of them received LT. In addition, our study defines disease course based on the changes in predicted FEV_1_ over time, because it is a quantifiable measure of changes in lung function that is readily applicable to clinical decision making^1^. There were irregularities in the frequency and interval of spirometry measurements among patients, which might explain some differences in our findings compared to those in other studies.

As mentioned above, the decision to use sirolimus seem discrepant from the recent clinical guidelines, due to a number of reasons. The late inclusion of sirolimus in the treatment of LAM for reimbursement by the Korean National Health Insurance may have contributed to the observed discrepancy. In addition, our study enrols patients diagnosed with LAM as early as 2005, when the benefit of mTOR inhibitors was not as clear as the current clinical recommendations.

Being a tertiary referral centre, we see a higher number of cases with severe disease, often resulting in LT. But the inclusion of a considerable proportion of LT cases also enables a more comprehensive overview of the clinical landscape of LAM. Moreover, the measurement of vascular endothelial growth factor-D, a potential biomarker for diagnosis and treatment response of LAM, were not available in our institution until recently (Supplementary Table S4).

In conclusion, sirolimus treatment predicts stable disease course in LAM patients. Better baseline function is also associated with stable disease course in LAM. A significant proportion of patients remain clinically stable even without treatment, highlighting the need for future studies identifying the clinical characteristics of this phenotype. We included a considerable proportion of patients undergoing LT, with better survival outcomes in more recent cases. Although highly heterogeneous in clinical presentation, LAM has a favourable overall prognosis. Survival rates in our study similar to reports in more recent studies.

## Methods

This study retrospectively reviewed the electronic medical records of patients diagnosed with LAM from 2005 to 2018 at Severance Hospital, South Korea. The anonymised data on clinical history, lab studies, PFTs, and imaging studies of the patients have been obtained for analysis. This study was approved by the Institutional Review Board (IRB) of Severance Hospital (IRB number: 4-2012-0685), which waived the requirement of obtaining written informed consent due to the retrospective nature of this study. The present study was conducted in accordance with the Declaration of Helsinki.

The diagnosis of LAM was confirmed according to the 2010 ERS criteria^1^. The diagnosis of TSC-LAM was made in patients with definite TSC, as defined by the TSC diagnostic criteria update in 2012^25^.

Statistical analysis was performed using SPSS software version 23 (www.ibm.com/analytics/spss-statistics-software). Mann–Whitney U test was used to compare median values between the groups. Fisher’s exact test and chi-square tests were performed to compare proportions between the groups. Logistic regression analysis was used to assess the impact of clinical features on the disease course. Variables included in the multivariate analyses either show significance level of *p* < 0.10 in univariate analysis, or is a PFT parameter, which reflects disease status in LAM. For all analyses, a two-tailed significance level of 0.05 was used. Kaplan–Meier analysis was performed for survival analysis.

## Supplementary Information

Supplementary Information.

## Data Availability

The datasets generated during and/or analysed during the current study are available from the corresponding author on reasonable request.
