# Supplementary material for: Long-term clinical course and progression of lymphangioleiomyomatosis in a single lung transplant referral centre in Korea
Source: Sci Rep. 2022 May 18;12:8260. doi: 10.1038/s41598-022-12314-1 (PMC9117329; doi:10.1038/s41598-022-12314-1)
Supplement: Supplementary file 1 — Supplementary Information. [file 41598_2022_12314_MOESM1_ESM.doc]

**Supplementary Figure S1.**

**
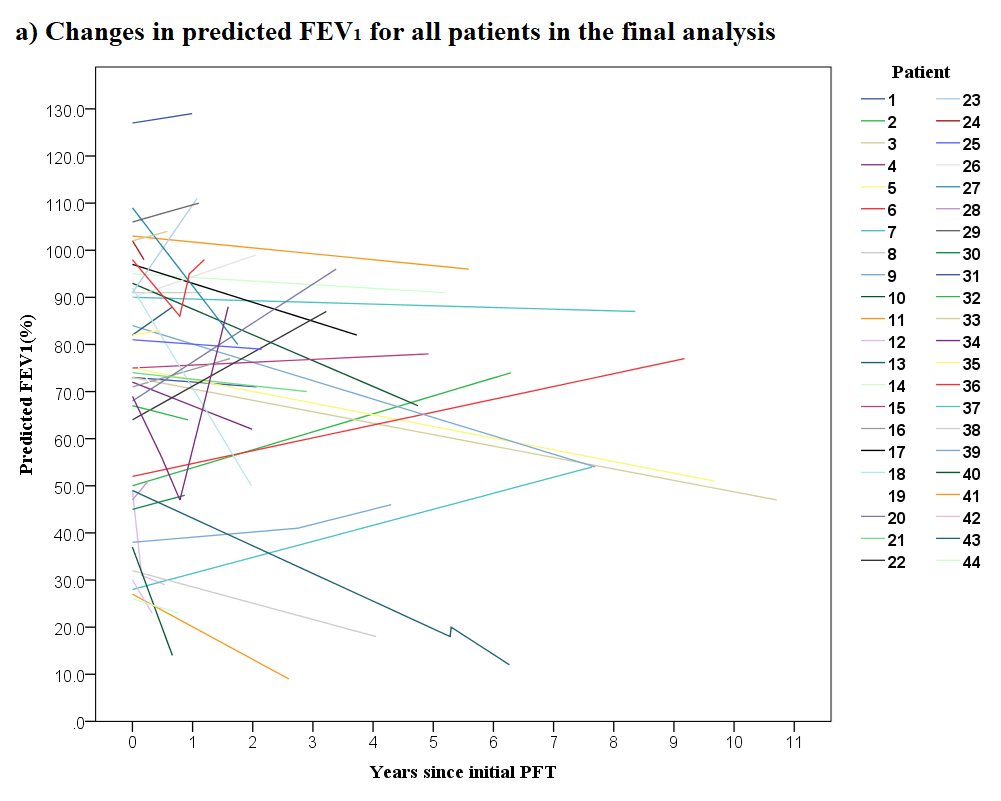
**

Changes in predicted FEV_1_ for all patients in the final analysis, with at least two sets of PFT results available (n=44), created using SPSS software version 23 (www.ibm.com/analytics/spss-statistics-software). For each patient, year 0 is the time when initial PFT was performed. *FEV_1_*, forced expiratory volume in 1 second; *PFT*, pulmonary function test.

**Supplementary Figure S1. (continued)**


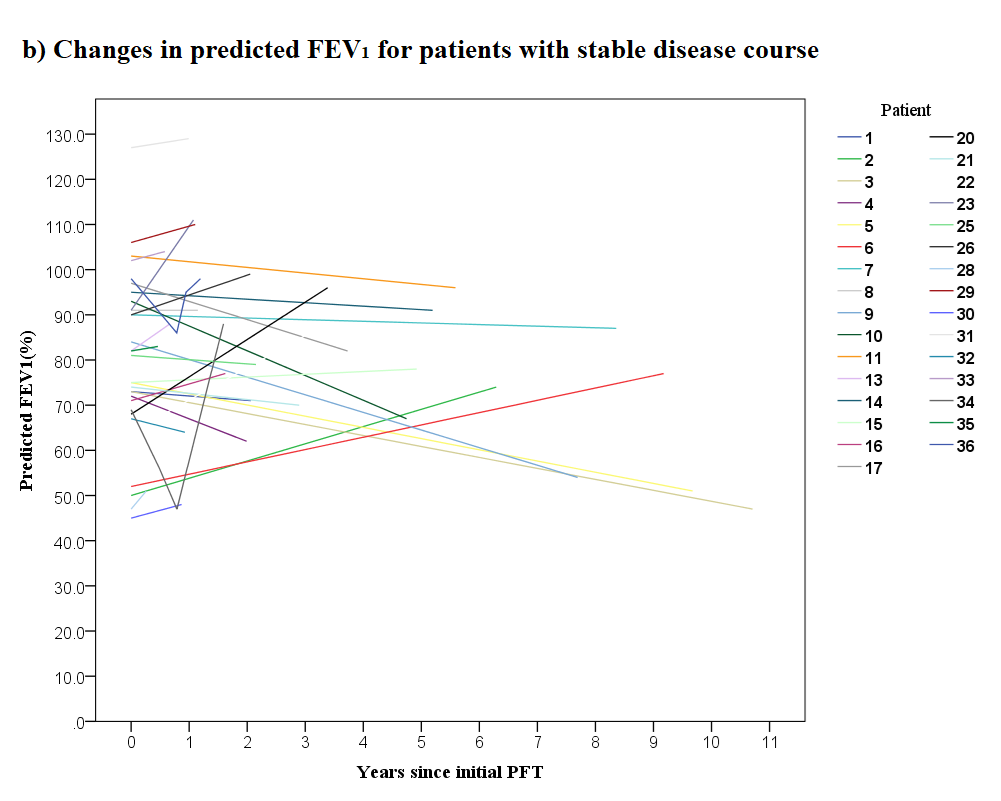


Changes in predicted FEV_1_ for patients with stable disease course, with at least two sets of PFT results available (n= 31), created using SPSS software version 23 (www.ibm.com/analytics/spss-statistics-software). For each patient, year 0 is the time when initial PFT was performed. The patient with the greatest annual decline in predicted FEV_1_ (patient #10) has a change of -5.48%/year. *FEV_1_*, forced expiratory volume in 1 second; *PFT*, pulmonary function test.

**Supplementary Figure S1. (continued)**


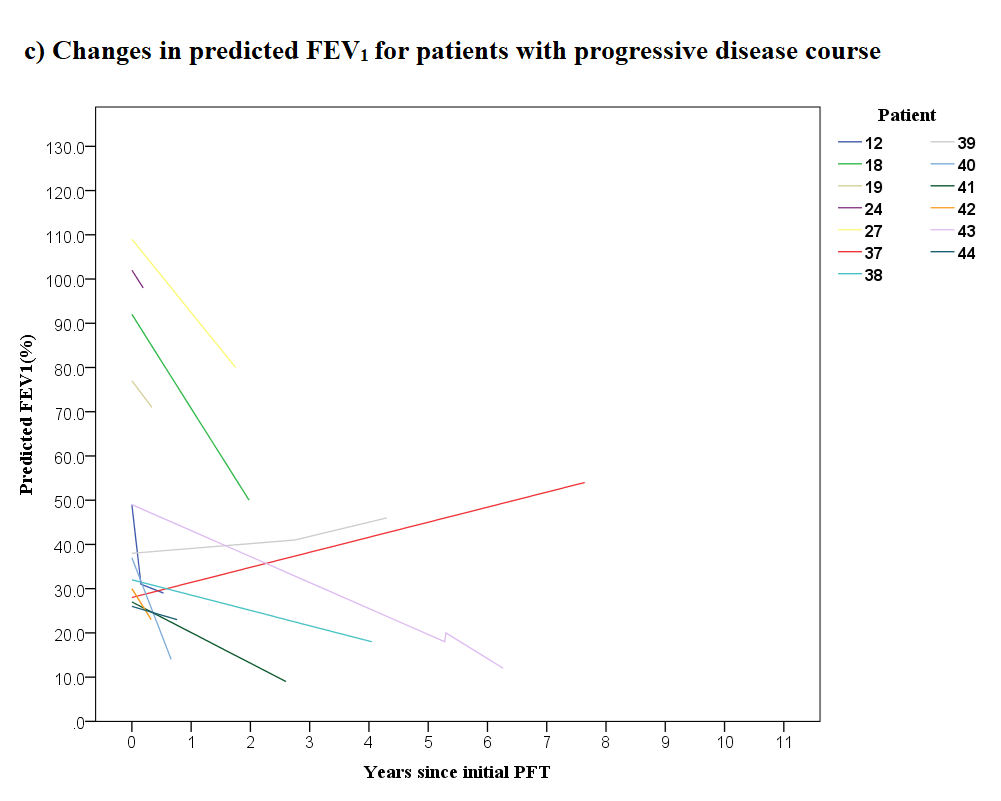


Changes in predicted FEV_1_ for patients with progressive disease course, with at least two sets of PFT results available (n= 13), created using SPSS software version 23 (www.ibm.com/analytics/spss-statistics-software). For each patient, year 0 is the time when initial PFT was performed. Two patients (#37, #39) who show increase in predicted FEV_1_ over time both received lung transplantation. In these patients, the initial PFT was performed before transplantation; subsequent PFT were performed after transplantation. *FEV_1_*, forced expiratory volume in 1 second; *PFT*, pulmonary function test.

**Supplementary Figure S2.**


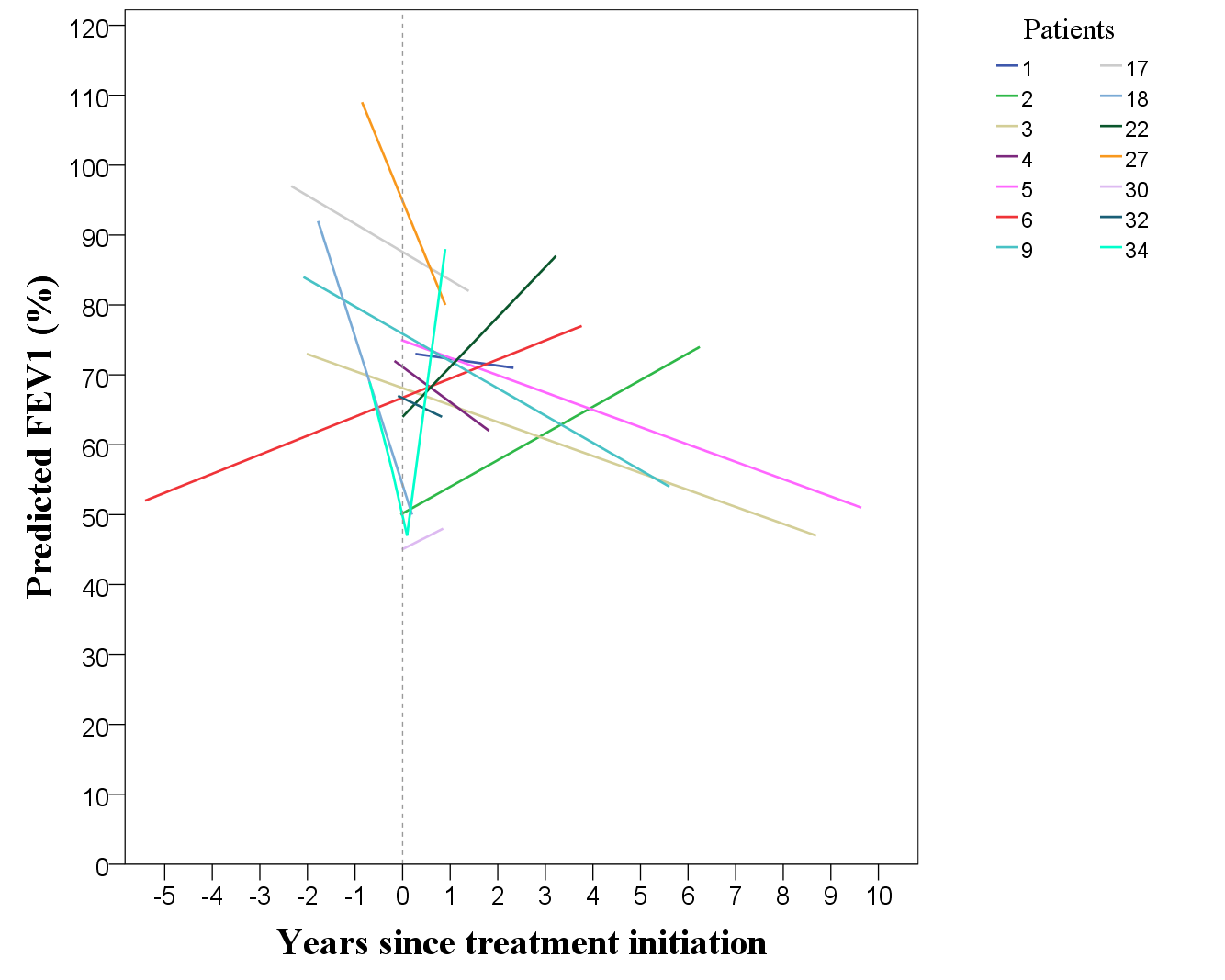


Changes in predicted FEV_1_ for patients treated with sirolimus, created using SPSS software version 23 (www.ibm.com/analytics/spss-statistics-software). Data are presented for patients (n=14) with at least one pulmonary function test available each for both before and after treatment initiation. For each patient, year 0 is the time when sirolimus treatment was initiated. *FEV_1_*, forced expiratory volume in 1 second.

**Supplementary Figure S3.**


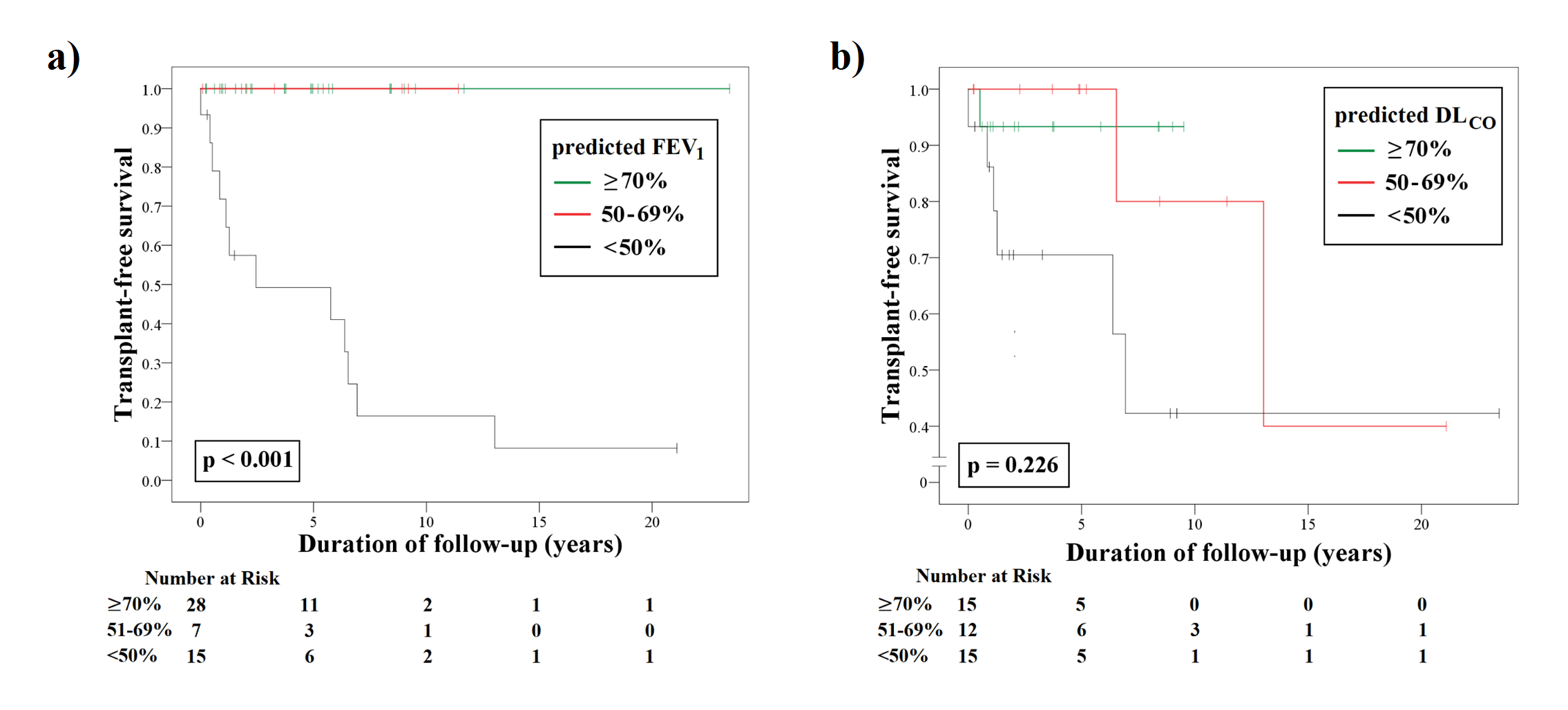


Kaplan-Meier analysis of transplant-free survival, after grouping patients based on a) baseline FEV_1_, and b) baseline DL_CO_. Baseline FEV_1_ of less than 50% predicted is associated with higher risk of progression to death or transplantation, while baseline DL_CO_ was not associated with increased risk of death or transplantation. Figure was created using SPSS software version 23 (www.ibm.com/analytics/spss-statistics-software) and Microsoft Paint version 11.2201. *FEV_1_*, forced expiratory volume in 1 second; *DL_CO_*, diffusion capacity of the lungs for carbon monoxide.

**Supplementary Figure S4.**


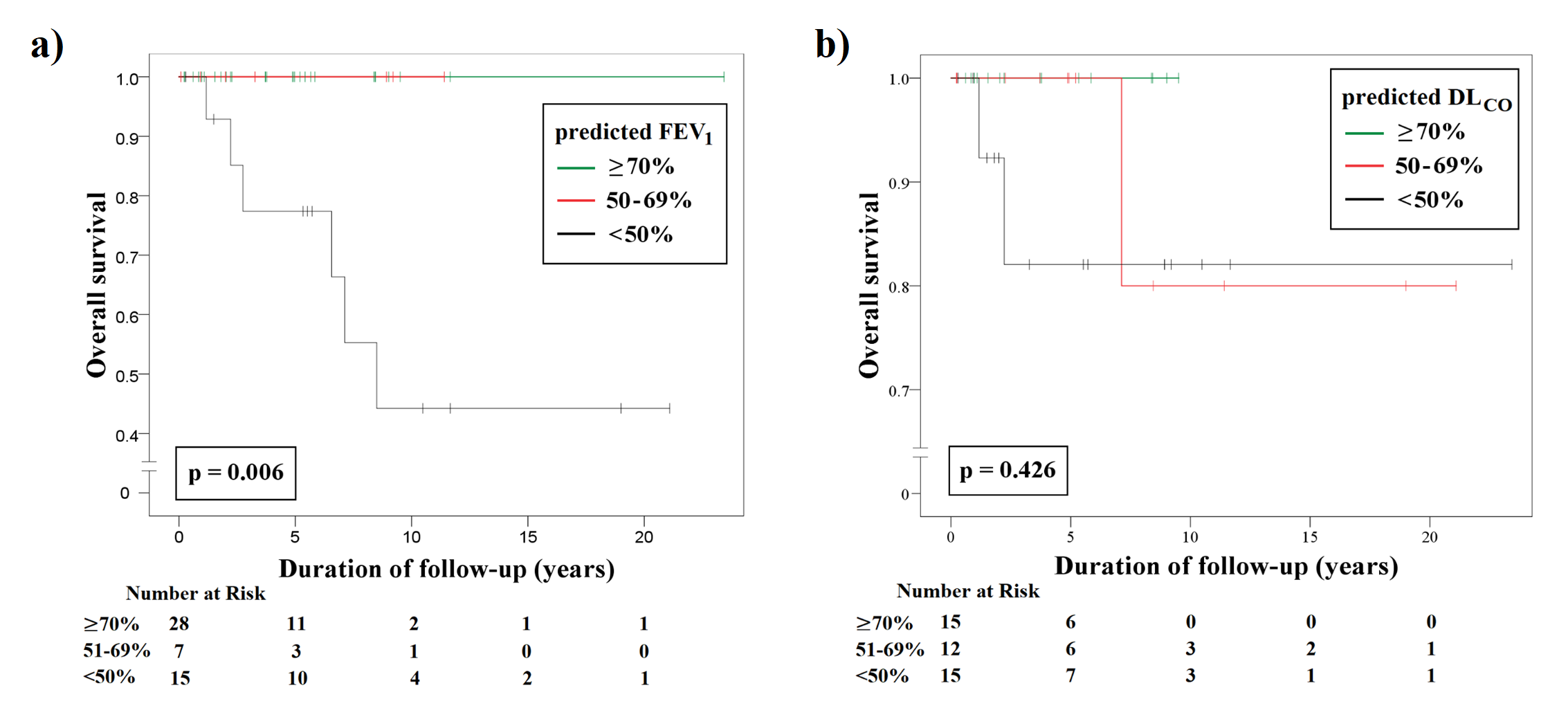


Kaplan-Meier analysis of overall survival, after grouping patients based on a) baseline FEV_1_, and b) baseline DL_CO_. Baseline FEV_1_ of less than 50% predicted is associated with higher risk of progression to death, while baseline DL_CO_ was not associated with increased risk of death. Figure was created using SPSS software version 23 (www.ibm.com/analytics/spss-statistics-software) and Microsoft Paint version 11.2201. *FEV_1_*, forced expiratory volume in 1 second; *DL_CO_*, diffusion capacity of the lungs for carbon monoxide.

**Supplementary Table S1.**

|  |  | **Univariate** | | |  | **Multivariate Model 1** | | |  | **Multivariate Model 2** | | |
| --- | --- | --- | --- | --- | --- | --- | --- | --- | --- | --- | --- | --- |
| **Parameters** |  | **OR** | **95% CI** | ***p-*value** |  | **OR** | **95% CI** | ***p-*value** |  | **OR** | **95% CI** | ***p-*value** |
| Age at diagnosis > 35years |  | 0.509 | 0.162-1.601 | 0.248 |  | - | - | - |  | - | - | - |
| Menopause |  | 0.933 | 0.087-10.040 | 0.955 |  | - | - | - |  | - | - | - |
| TSC-LAM |  | 0.625 | 0.113-3.461 | 0.590 |  | - | - | - |  | - | - | - |
| Use of sirolimus |  | 0.224 | 0.055-0.908 | **0.036** |  | 0.176 | 0.031-0.982 | **0.048** |  | 0.118 | 0.023-0.602 | **0.010** |
| FVC, ≤80% pred. |  | 2.587 | 0.773-8.656 | 0.123 |  | 0.781 | 0.119-5.128 | 0.797 |  | - | - | - |
| FEV_1_, ≤80% pred. |  | 4.958 | 1.197-20.546 | **0.027** |  | 4.785 | 0.701-32.657 | 0.110 |  | 5.026 | 1.021-24.741 | **0.047** |
| FEV_1_/FVC, ≤70% pred. |  | 17.333 | 3.866-77.719 | **<0.001** |  | - | - | - |  | - | - | - |
| DL_CO_, ≤80% pred. |  | 5.200 | 0.580-46.602 | 0.141 |  | 3.036 | 0.278-33.106 | 0.362 |  | - | - | - |
| Pneumothorax |  | 1.000 | 0.301-3.321 | 1.000 |  | - | - | - |  | - | - | - |
| Abdominal lymphadenopathy |  | 2.800 | 0.858-9.139 | **0.088** |  | 1.916 | 0.377-9.738 | 0.433 |  | 2.556 | 0.573-11.412 | 0.219 |
| Angiomyolipoma |  | 0.280 | 0.069-1.142 | **0.076** |  | 0.461 | 0.078-2.716 | 0.392 |  | 0.426 | 0.082-2.207 | 0.309 |
| Chylothorax |  | 1.000 | 0.165-6.052 | 1.000 |  | - | - | - |  | - | - | - |

Logistic regression analysis of factors associated with the progression of LAM, using alternate cutoffs for PFT parameters. Progression of LAM is defined as receiving lung transplantation or ΔFEV_1_ less than -10%/year, as described in Figure 1. All data for PFT parameters use values at baseline PFT parameters are categorical variables, with 80% of predicted value for FVC, FEV_1_, and DL_CO_, and 70% of predicted value for FEV_1_/FVC ratio as cut-offs. The following variables are included in each model for multivariate analysis: use of sirolimus, predicted FVC, predicted FEV_1_, predicted DL_CO_, abdominal lymphadenopathy, and angiomyolipoma for model 1; use of sirolimus, predicted FEV_1_, abdominal lymphadenopathy, and angiomyolipoma for model 2. *OR*, odds ratio; *CI*, confidence interval; *TSC*, tuberous sclerosis complex; *LAM*, lymphangioleiomyomatosis; *mTOR*, mechanistic target of rapamycin; *FVC*, forced vital capacity; *FEV_1_*, forced expiratory volume in 1 second; *DL_CO_*, diffusion capacity of the lungs for carbon monoxide; *PFT*, pulmonary function test.

**Supplementary Table S2.**

| **Profile variables** | | **Value** |
| --- | --- | --- |
| **Any event of mTOR inhibitor discontinuation, n (%)** | | 12 (52.2) |
|  | Permanent | 7 (30.4) |
|  | Intermittent | 5 (21.7) |
| **Adverse events, n (%)** | | 14 (60.9) |
|  | Oral mucositis/ulcer | 8 (34.8) |
|  | Infection | 4 (17.4) |
|  | Gastrointestinal (dyspepsia, diarrhea, discomfort) | 3 (13.0) |
|  | Neutropenia | 1 (4.3) |
|  | Ocular symptom (blurry vision) | 1 (4.3) |
|  | Skin manifestation | 1 (4.3) |
| **Sirolimus dosage, mean ± standard deviation** | |  |
|  | Treatment duration (months)^*^ | 27.6 ± 24.7 |
|  | Average daily dose (mg/day)^ǂ^ | 1.80 ± 0.62 |
|  | Average drug level (ng/mL)^§^ | 7.96 ± 2.53 |

Treatment profiles of patients initially treated with mTOR inhibitors. Data available in ^*^*n=*16, ^ǂ^*n=*17, ^§^*n=*15 patients, respectively. *mTOR*, mechanistic target of rapamycin.

**Supplementary Table S3.**

|  |  |  | **mTOR inhibitor (*n=*23)** | | |  | **Observation (*n=*31)** | | |
| --- | --- | --- | --- | --- | --- | --- | --- | --- | --- |
|  |  |  | **Stable (*n=*19)** | **Progressive (*n=*4)** | ***p-*value** |  | **Stable (*n=*17)** | **Progressive (*n=*14)** | ***p-*value** |
| Females | |  | 19 (100.0) | 4 (100.0) | - |  | 16 (94.1) | 14 (100.0) | 1.000 |
| Age at diagnosis, years | |  | 33.00 [39.00-4.00] | 33.00 [23.75-34.75] | 0.409 |  | 42.00 [36.50-47.00] | 36.50 [30.75-41.25] | 0.109 |
| Menopause at diagnosis | |  | 0 (0.0) | 0 (0.0) | - |  | 3 (17.6) | 1 (7.1) | 0.602 |
| Median follow-up, years | |  | 3.78 [8.92-4.00] | 1.05 [0.26-3.23] | 0.097 |  | 2.22 [1.03-7.11] | 6.74 [4.69-10.78] | **0.015** |
| Lung transplantation | |  | 0 (0.0) | 0 (0.0) | - |  | 0 (0.0) | 13 (92.9) | **<0.001** |
| Mortality | |  | 0 (0.0) | 0 (0.0) | - |  | 0 (0.0) | 6 (42.9) | **0.004** |
| **Diagnosis** | |  |  |  | 1.000 |  |  |  | 1.000 |
|  | Probable |  | 1 (5.3) | 0 (0.0) |  |  | 1 (5.9) | 0 (0.0) |  |
|  | Definite |  | 18 (94.7) | 4 (100.0) |  |  | 16 (94.1) | 14 (100.0) |  |
|  | Biopsy-proven |  | 10 (52.6) | 1 (25.0) | 0.590 |  | 11 (64.7) | 14 (100.0) | **0.021** |
| **Type of LAM** | |  |  |  | 0.194 |  |  |  | 1.000 |
|  | TSC-LAM |  | 3 (15.8) | 2 (50.0) |  |  | 1 (5.9) | 0 (0.0) |  |
|  | Sporadic LAM |  | 16 (84.2) | 2 (50.0) |  |  | 16 (94.1) | 14 (100.0) |  |
| Characteristic HRCT | |  | 19 (100.0) | 4 (100.0) | - |  | 17 (100.0) | 14 (100.0) | - |
| Pneumothorax | |  | 15 (78.9) | 2 (50.0) | 0.270 |  | 9 (52.9) | 10 (71.4) | 0.461 |
| Abdominal LAP | |  | 11 (57.9) | 2 (50.0) | 1.000 |  | 4 (23.5) | 10 (71.4) | **0.012** |
| Angiomyolipoma | |  | 8 (42.1) | 1 (25.0) | 1.000 |  | 7 (41.2) | 2 (14.3) | 0.132 |
| Chylothorax | |  | 4 (21.1) | 1 (25.0) | 1.000 |  | 0 (0.0) | 1 (7.1) | 0.452 |
| **Baseline PFT** | |  |  |  |  |  |  |  |  |
| FVC, % predicted | |  | 81.50 [87.75-4.00]^*^ | 90.00 [84.50-107.50] | 0.053 |  | 90.00 [83.00-95.00]^§^ | 57.00 [36.50-87.00]^**^ | **0.013** |
| FEV_1_, % predicted | |  | 72.50 [82.50-4.00]^*^ | 97.00 [59.75-107.25] | 0.141 |  | 91.00 [81.00-102.00]^§^ | 28.00 [15.50-37.50]^**^ | **<0.001** |
| FEV_1_/FVC, % predicted | |  | 72.50 [82.50-4.00]^*^ | 81.50 [55.75-88.50] | 0.434 |  | 82.00 [74.00-85.00]^§^ | 36.00 [30.00-46.00]^**^ | **<0.001** |
| DL_CO_, % predicted | |  | 53.50 [74.00-4.00]^ǂ^ | 54.50 [25.50-64.00] | 0.798 |  | 78.00 [69.50-86.00]^ǂ^ | 27.50 [14.75-52.50]^ǂǂ^ | **0.001** |

Subgroup analysis of characteristics of disease progression by mTOR inhibitor use. Patients in the same treatment group were analyzed by disease course. All data are expressed in n (%) or median [interquartile range]. Data for DL_CO_ were available for ^*^*n=*18, ^ǂ^ *n=*14, § *n=*15, ^**^*n=*13, ^ǂǂ^*n=*10 patients. *mTOR*, mechanistic target of rapamycin; *LAM*, lymphangioleiomyomatosis; *TSC*, tuberous sclerosis complex; *HRCT*, high-resolution computed tomography; *LAP*, lymphadenopathy; *PFT*, pulmonary function test; *FVC*, forced vital capacity; *FEV_1_*, forced expiratory volume in 1 second; *DL_CO_*, diffusion capacity of the lungs for carbon monoxide.

**Supplementary Table S4.**

|  | **All (n=7)** | **Stable (n=5)** | **Progressive (n=2)** | ***p*-value** |
| --- | --- | --- | --- | --- |
| VEGF-D | 520.50 [351.05-1214.70] | 396.82 [351.06-796.60] | 994.26 [520.50-1468.02] | 0.381 |
| (pg/mL) |

VEGF-D measurements in patients with LAM. All data expressed in median [interquartile range]. These measurements were performed on an average of 9.85 years after diagnosis of LAM. *VEGF-D*, vascular endothelial growth factor-D; *LAM*, lymphangioleiomyomatosis.
